# A lethal synergy induced by phellinus linteus and camptothecin11 in colon cancer cells

**DOI:** 10.18632/oncotarget.23918

**Published:** 2018-01-04

**Authors:** Tianqi Yu, Suthakar Ganapathy, Ling Shen, Bo Peng, Sung-Hoon Kim, Alexandros Makriyannis, Changyan Chen

**Affiliations:** ^1^ The Center for Drug Discovery, Northeastern University, Boston, MA, USA; ^2^ Cancer Molecular Targeted Herbal Research Center, College of Korean Medicine, Kyung Hee University, Seoul, Republic of Korea

**Keywords:** PLGL, CPT11, Chk1, cyclin E, S phase

## Abstract

Side effects of anti-cancer drugs are always challenging for effective cancer treatments. The polysaccharides extracted from *Phellinus linteus* (PLGL) have been widely used in treating cancers. However, the mechanism by which PLGL antagonizes cancerous growth has not been fully investigated. The current study demonstrated that human colon cancer HCT116 and HT29 cells became highly susceptible to cell death when being co-treated with PLGL and low dose of camptothecin11 (CPT11, a topoisomerase inhibitor-based drug), the efficacy of which was comparable as that generated by the high dose of CPT11. However, the co-treatment, unlike high doses of CPT11, was not cytotoxic to the control immortalized colon Caco-2 cells. The co-treatment caused high percentages of the colon cancer cells to accumulate in S phase of the cell cycle, which was also seen in the same cells received the high dose of CPT11 treatment. Chk1 was phosphorylated, and then rapidly degraded in the cancer cells treated with the high dose of CPT11 or co-treatment, but not in the cells treated with PLGL alone or low doses of CPT11. PLGL appeared enhancing CPT11 inhibitory effect on topoisomerase, and Chk1 degradatopm in the cancer cells. Furthermore, cyclin E (clnE) became unstable at the transcription level in co-treated or PLGL-treated colon cancer cells. The data suggested that PLGL functions in two ways to achieve its lethal synergy with CPT11 in colon cancer cells. Our findings are of potential significance as PLGL represents a promising medicine for overcoming the side effects of CPT11 and perhaps also for improving other CPTs-based regimens.

## INTRODUCTION

Colon malignancy is the second leading cause of cancer mortality worldwide [1–4]. A colon cancer cell evolving from colon epithelium usually undergoes a predictable progression of histological alterations and, concurrent genetic and epigenetic changes, which provide a growth advantage for oligo-clonal expansions from pre-malignant stages to cancer. The earliest recognisable lesions in sporadic colon cancer formation seem to be aberrant crypt foci that subsequently progress to adenomas and adenocarcinomas. Sporadic colon cancer is initiated by changes in Wingless (Wnt)-regulated signaling pathways, which permit activation of oncogenes or loss of function of tumor suppressors. Genes mutated or deleted during colon tumorigenesis consist of *B-raf*, *K-ras* or *p53* [5–8]. Upon oncogenic activation of K-Ras or B-Raf, several intracellular growth-related signalling pathways are upregulated, resulting in perturbation of cell cycle checkpoints or enhance of pro-survival activities. Together, multiple changes at genetic and epigenetic levels are in favour of the adenomas to undergo transformation. The prognosis of advanced colon cancer is dismal, and thus, better therapeutics is urgently needed.

*Phellinus linteus* (PL) is an Asian medicinal fungus and has been using in many Asian countries to boost human health as well as treat human malignant diseases, including colon cancer [8–14]. PL consists of various bio-active substances that possess complicated chemical natures. Through a combination of ethanol precipitation, fractional concentration, gel filtration and biological evaluations, the polysaccharides are proven to be the main active components (PLGL) for its anti-cancer activity [15, 16]. Studies demonstrated that PLGL can boost human immune system, through improving antigen presentation and increasing the expression of cell surface markers (for example, MHC I/II) to promote dendritic cell migration into lymphoid tissues [10, 11, 14]. PLGL treatment also enhances B lymphocyte activities. We demonstrated that PLGL at high doses (> 1 mg/ml) sensitized several types of cancer cells to apoptosis, but had insignificantly harmful impact on normal cells or surrounding tissues [17, 18]. In this apoptotic process, the G_1_ and S checkpoints were activated and responsible for killing the cancer cells.

CPT11 is a topoisomerase inhibitor-based drug that blocks DNA unwinding in S phase of the cell cycle when replication, transcription and chromatin remodeling are taken place. Cells death triggered by CPT11 often also occurs in S phase, via small interfering RNA-mediated depletion of the checkpoint kinase 1 (Chk1) [19–21]. However, this drug is relatively toxic and possesses strong side effects (such as lowing blood counts and causing severe body responses at conventional treatment doses).

Chk1 and 2 are checkpoint regulators and phosphorylated by ATM/ATR in response to DNA replication or damage stresses [22–24]. ATR/Chk1 signaling is activated by a broader spectrum of genotoxic stimuli. The phosphorylated Chk1 has different functions. For example, its phosphorylation at ser-317 or ser-345 residue is necessary for ensuring proper G_1_/S transition [25, 26]. Chk1 degradation is through ubiquitination. A timingly proper coupling activation and destruction prevents Chk1 accumulation, leading to a successful S phase transition. Genotoxic stress often activates Chk1, which is able to stabilize stalled or aberrant replicative structures of DNAs for damage repair. Loss of Chk1 triggers the accumulation of cells in S phase of the cell cycle, resulted in the formation of aberrant chromosomal structures. If damages are overwhelming or persistent, an apoptotic crisis occurs. Many anti-cancer drugs (such as CPT11) target Chk1 to sensitize cancer cells for the induction of apoptosis.

Cyclins (clns) D, E and A are the important cell cycle regulators in the G_1_ or S phases, via regulating the activities of CDKs. The S phase transition in cell cycle progression was primarily regulated by the clnE/CDK2 complex [27, 28]. Although clnD was also involved in the G_1_/S transition, all phenotypic and developmental defects in mice caused by clnD deficiency could be rescued by *clnE* knock-in at the *clnD1* locus, suggesting that the function of clnD1 can be replaced by clnE [29, 30]. ClnE expression oscillated during cell cycle progression, which was tightly regulated at transcriptional and post-transcriptional levels [27, 28].

In this study, we demonstrated that PLGL acted in synergy with the low dose of CPT11 to achieve an effective killing of colon cancer cells. In response to the co-treatment of PLGL and CPT11, a rapid loss of Chk1 protein as well as of *clnE* occurred in the colon cancer cells. Subsequently, the cancer cells were accumulated in S phase of the cell cycle, leading to apoptosis. Thus, our findings suggested that PLGL could be a promising therapeutic compound for improving the efficacy of CPT-based regimens.

## RESULTS

### PLGL was in synergy with the low dose of CPT11 for triggering apoptosis in cultured and xenografted colon cancer

**Figure 1:**
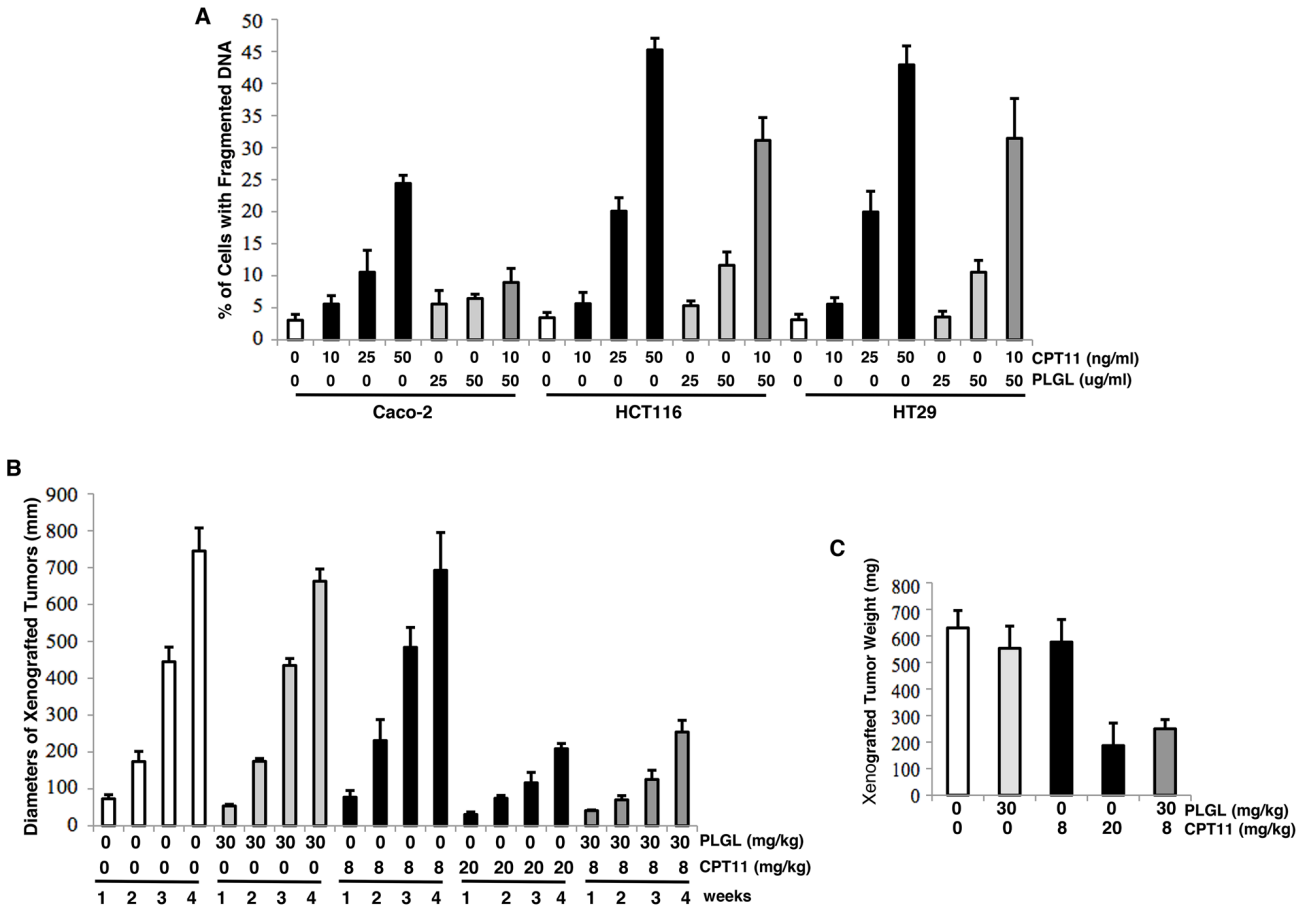
Induction of the lethal synergy by the co-treatment of PLGL and CPT11 in colon cancer cells *in vitro* and *in vivo* **(A)** The colon immortalized Caco-2 or cancer HCT116 and HT29 cells were treated with different dosed of CPT11, PLGL or both for 48 h. Subsequently, the induction of apoptosis was analyzed by DNA fragmentation assay. The error bars represent the standard deviations (SD) from 5 independent experiments (*p*< 0.01). **(B)** Cells were inoculated subcutaneously into the nude mice. CPT11, PLG or both was injected intraperitoneally after the inoculation and subsequently administrated every 4 days. One week later, the diameters of the tumors were measured weekly for consecutive 4 weeks. The measurements were plotted. The error bars represent the SD (*p* <0.05). **(C)** At the end of the experiments (4 weeks), the mice were sacrificed, the tumors were weighted and the measurements were plotted. The error bars represent the SD (*p* <0.05).

Studies indicated that PLGL perturbs cell cycle restrictions (mainly on G_1_/S phases) and induces apoptosis in several different types of cancer cells [16, 17, 31–33]. CPT11 is known to via inhibiting topoisomerase, kill cancer cells, especially colon cancer cells [19–21]. Therefore, we tested the effect of the combination treatment of PLGL with CPT11 on colon cancer cells. DNA fragmentation assay was conducted after human colon immortal Caco-2 and malignant HTC116 or HT29 cells were treated with CPT11, PLGL or both at different concentrations for 48 h (Figure 1A). CPT11 at 25 ng/ml were cytotoxic to the cancer cells and the toxicity was increased in a dose-dependent fashion. In comparison, this drug appeared slightly less toxic to Caco-2 cells. PLGL at the doses being tested did not have obvious cytotoxic effect on Caco-2 cells and very low percentages of HCT116 or HT29 cells appeared sensitive to 50 ug/ml of PLGL. When being co-treated with PLGL (50 ug/ml) and CPT (10 ng/ml) for 48 h, approximately 35% of the colon cancer cells become apoptotic, but the co-treatment was not apoptotic to Caco-2 cells. The similar results were obtained from Annexin V analysis (data not shown). The results suggested that the combination treatment of PLGL and low dose of CPT11 acted in synergy for killing colon cancer cells.

To further determine of the effect of the lethal synergy induced by the co-treatment of PLGL and CPT11, xenograft assay was performed. After inoculating HCT116 cells into nude mice, CPT11, PLGL and both were intraperitoneally injected into the mice, respectively [34, 35]. The injections were repeated every 4 days. One week later when the tumors became detectable, the diameters of the tumors were measure every week for consecutive 4 weeks (Figure 1B). The xenografted tumors were formed and grown in the mice untreated and treated with the low dose of CPT11 or PLGL alone. However, the sizes of the tumors in the mice received the co-treatment of PLGL plus the low dose of CPT11 grew as slower as those injected with the high dose of CPT11. At the end of the experiments (4 weeks), the mice were sacrificed, the tumors were weighted and the measurements were plotted (Figure 1C). The *in vivo* data appeared in a good agreement with the *in vitro* results.

### Colon cancer cells were accumulated in S phase of the cell cycle after the co-treatment of PLGL and CPT11

**Figure 2:**
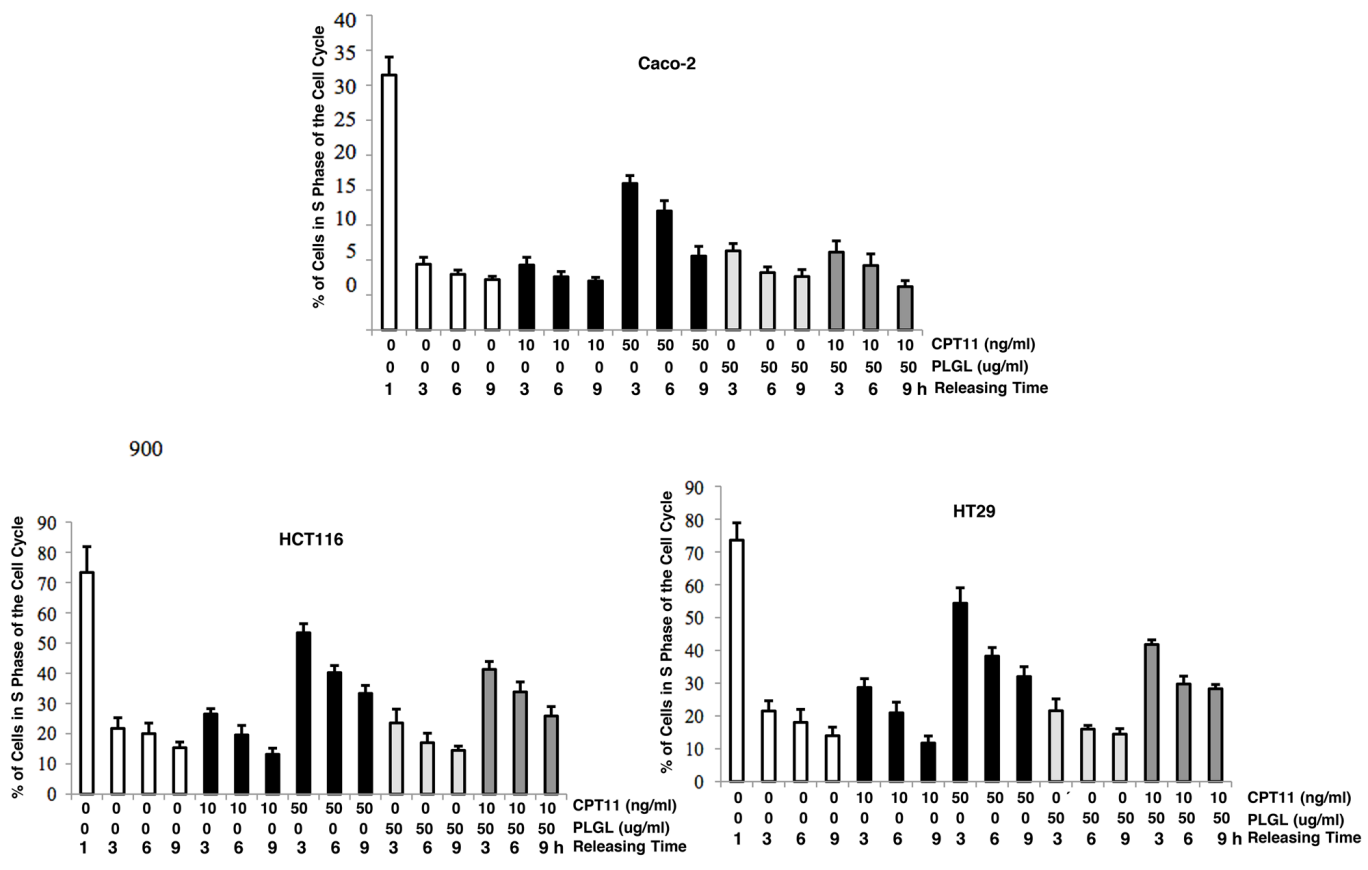
Colon cancer cells accumulated in S phase in response to the co-treatment The cells were treated with PLGL, CPT11, or both prior to thymidine synchronization and cell cycle progression was analyzed at different time points after released from thymidine blockade. Percentages of cells in the S phase were plotted. Error bars are SD over 5 experiments (*p*<0.05).

As a topoisomerase inhibitor, CPT11 blocks DNA unwinding, leading to S phase arrest and further apoptosis [19–21]. During conducting DNA fragmentation assay, we noticed the S phase accumulation profile in the co-treated colon cancer cells before apoptosis occurred. Therefore, the DNA profiles of the cells in response to the high dose of CPT11 (50 ng/ml), PLGL or co-treatment were analyzed by flowcytometry (Figure 2). Prior to the treatments, the cells were synchronized at the boundary of G_1_/S phases of the cell cycle by the double thymidine block. There were more than 70% of untreated or treated cells accumulated in the S phase 1 h after being released from the block. The untreated cells rapidly exited S phase 3 h after releasing from thymidine block, and approximately 15% of the cells remained in S phase at the latest testing time point. However, 3 h after the releasing, the majority (more than 55%) of the cancer cells and approximately 45% of Caco-2 cells, after the high dose of CPT11 (50 ng/ml) treatment, still remained in S phase, and the exiting kinetics from S phase were very slow at testing time points. The similar patterns of S phase accumulation were seen in the colon cancer cells co-treated with PLGL (50 ug/ml) and CPT11 (10 ng/ml), as that treated with the high dose of CPT11. Furthermore, the treatments of PLGL (50 ug/ml) and CPT11 (10 ng/ml) alone did not induce obvious S phase accumulations in all three cell lines. The results suggested that PLGL treatment acted in synergy with the low dose of CPT11 to block the cancer cells exiting from S phase.

### Chk1 was phosphorylated, but rapidly degraded in colon cancer cells after the co-treatment of PLGL and CPT11

**Figure 3:**
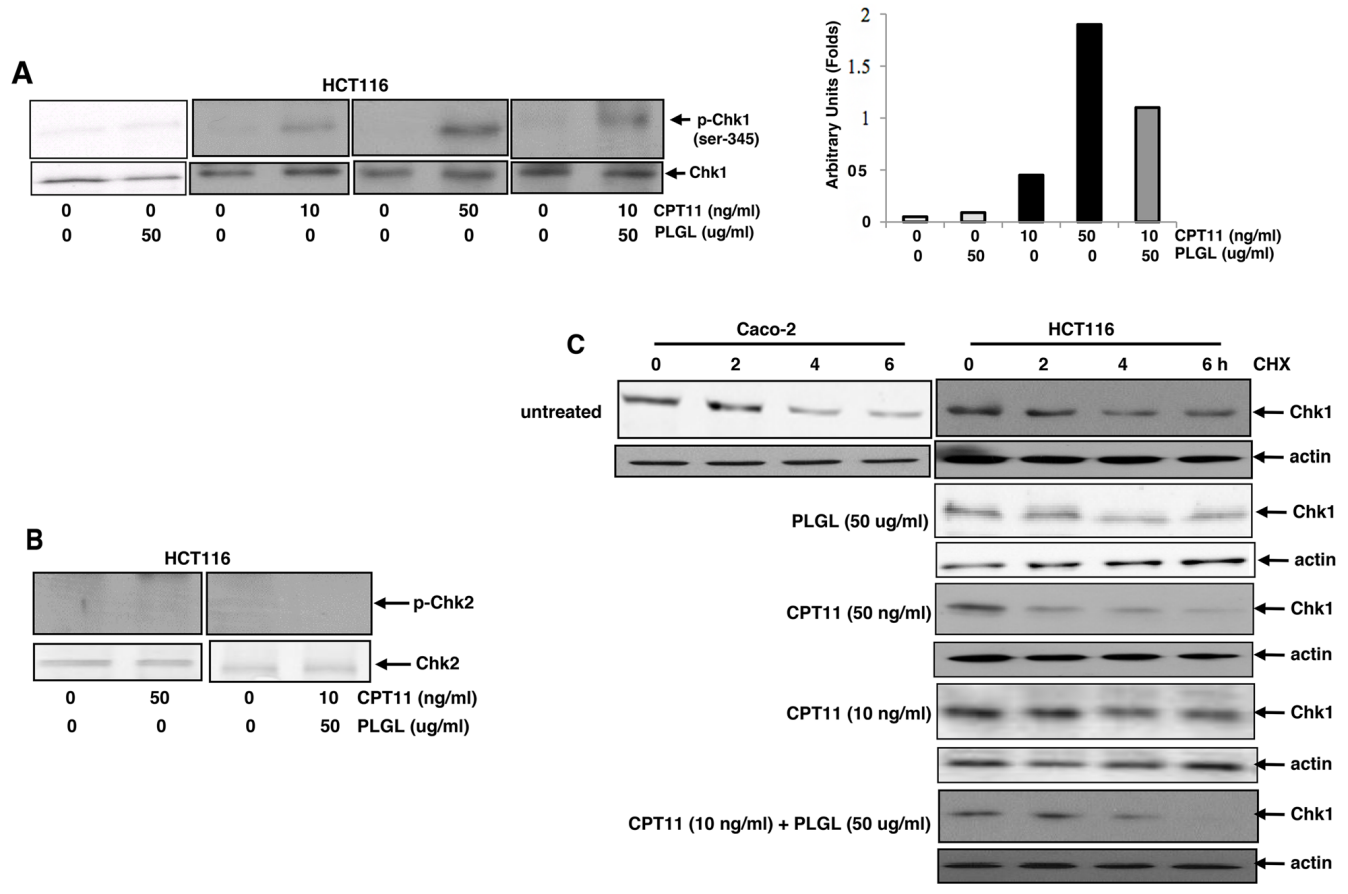
Chk1 was phosphorylated and rapidly degraded in co-treated colon cancer cells **(A)** After the treatments, Chk1 phosphorylation was analyzed by immunoblotting in HCT116 cell (left panels). The increasing folds of Chk1 phosphorylation were plotted (right panel). **(B)** Chk2 phosphorylation in HCT116 cells with or without the treatments was tested by immunoblotting. **(C)** After exposure to CHX, cell lysates from Caco-2 and HCT116 cells with or without the treatments at different time points were prepared and then subjected to immunoblotting to detect Chk1 degradation.

Chk1 is a checkpoint regulator and plays a crucial role in the regulation of the transitions of the G_1_ and S phases [35–39]. Via causing rapid Chk1 degradation and S phase crisis, CPT11 functions to eliminate cancer cells [19, 40, 41]. The activation of Chk1 at serine-345 is required for its activation and degradation [40–43]. Therefore, the phosphorylation of the ser-345 of Chk1 was examined in HCT116 cells following the treatment of CPT11 at different doses or its co-treatment with PLGL by immunoblot analysis (Figure 3A). A slight increase of phosphorylated Chk1 was detected in the cells treated with 10 ng/ml of CPT11, which was significantly upregulated by the high dose (50 ng/ml) of the drug. The co-treatment of CPT11 (10 ng/ml) and PLGL (50 ug/ml) also elevated the level of Chk1 phosphorylation in the cancer cells. The phosphorylated Chk1 was undetectable in the cells treated with PLGL alone. Chk2 phosphorylation status in the cells was then analyzed (Figure 3B). This cell cycle checkpoint regulator was not activated by the high dose of CPT11 or the co-treatment with PLGL. The results again indicated that PLGL was able to upregulate the activity of the low dose of CPT11 in the promotion of Chk1 phosphorylation in the colon cancer cells.

Next, we tested Chk1 stability in response to the co-treatment of CPT11 and PLGL. Caco-2 and HCT116 cells were treated with different doses of CPT11, PLGL or both (Figure 3C). After blocked protein synthesis by cycloheximid (CHX), the levels of Chk1 expression at different time points of the blocking were examined by immunoblotting. The kinetics of Chk1 degradation was represented in untreated Caco-2 and HCT116 cells, in which Chk1 started to degrade at 4 h after the block of the protein synthesis and could still be detected at 6 h of the blocking. In contrast, Chk1 was rapidly degraded in HCT116 cells treated with 50 ng/ml of CPT11 or its co-treatment with PLGL. PLGL treatment alone did not change the pattern of Chk1 degradation. The stability of *Chk1* at the post-transcriptional level was also examined by RT-PCR. The treatments of CPT11 or its co-treatment with PLGL did not alter *Chk1* stability in the colon cancer cells (data not shown). The results further implicated that PLGL might enhance the topoisomerase inhibitory activity of CPT11 for triggering premature depletion of Chk1 in colon cancer cells.

### Ectopic expression of Chk1 desensitized colon cancer cells to apoptosis induced by the co-treatment

**Figure 4:**
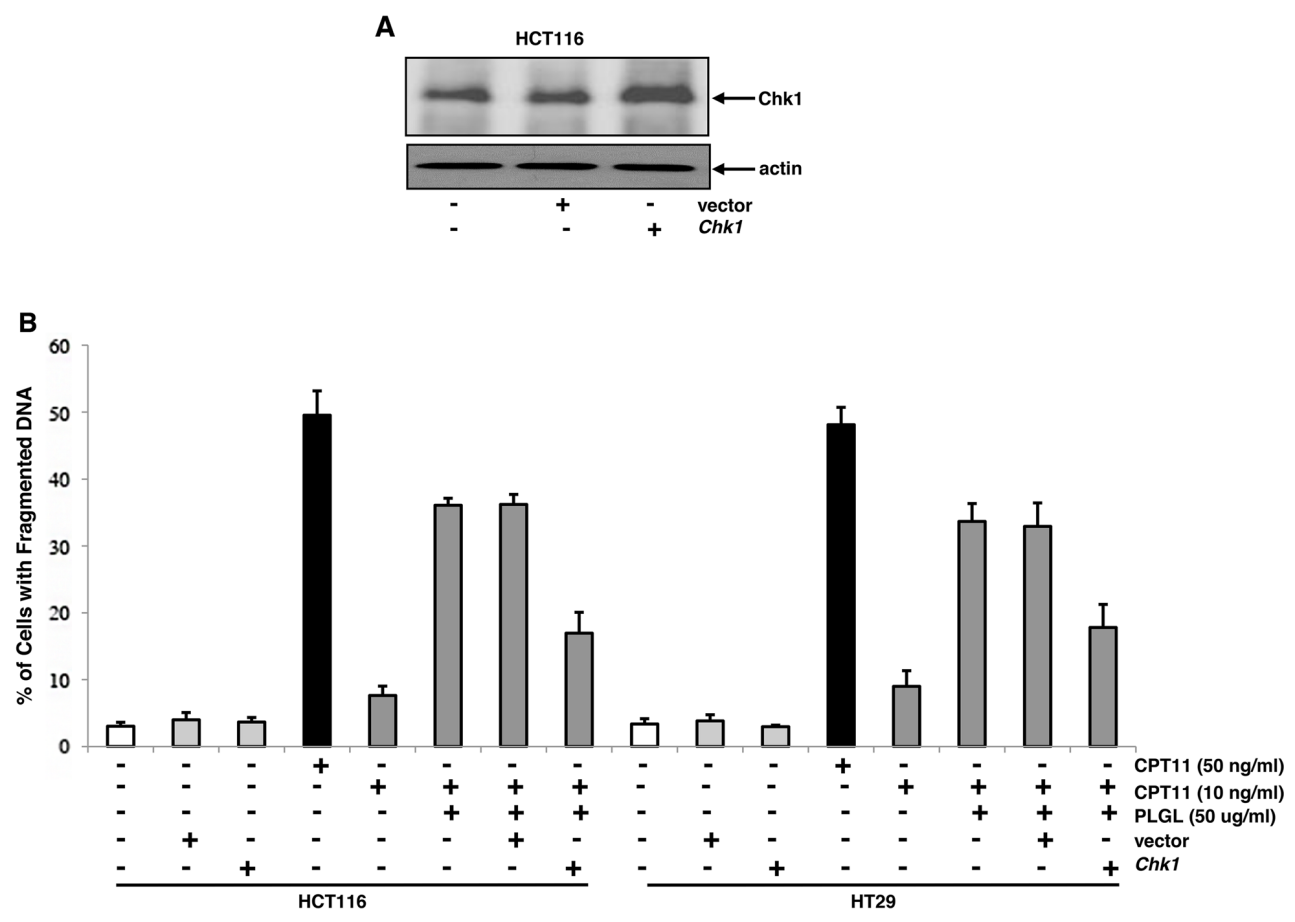
Overexpression of Chk1 partially desensitized the colon cancer cells to apoptosis induced by the co-treatment **(A)** *Chk1* was introduced into HCT116 cells and the level of Chk1 protein expression was determined by immunoblotting. **(B)** After the transfection of *Chk1*, the colon cancer cells were subjected to different treatments for 48 h. Subsequently, DNA fragmentation assay was performed to detect the induction of apoptosis. Error bars are SD over 5 independent experiments (*p*<0.005).

To further determine the importance of an unstable Chk1 in this lethal synergy, HCT116 cells were transfected with *Chk1*, the expression of which was analyzed by immunoblotting (Figure 4A). Subsequently, the induction of apoptosis was examined in colon cancer HCT116 and HT29 cells with or without overexpressing *Chk1* in response to different treatments (Figure 4B). The introduction of the vector or *Chk1* alone did not induce apoptosis in the colon cancer cells. After ectopic expression of *Chk1*, the cancer cells became partially insensitive to the co-treatment of PLGL and CPT11 to apoptosis. It indicates that Chk1 is a key element in the lethal synergy induced by the co-treatment. However, the overexpression of *Chk1* was unable to completely suppress apoptosis, indicating other factor(s) is/are involved in this process.

### Cyclin E became unstable at the transcriptional level in PLGL-treated colon cancer cells

**Figure 5:**
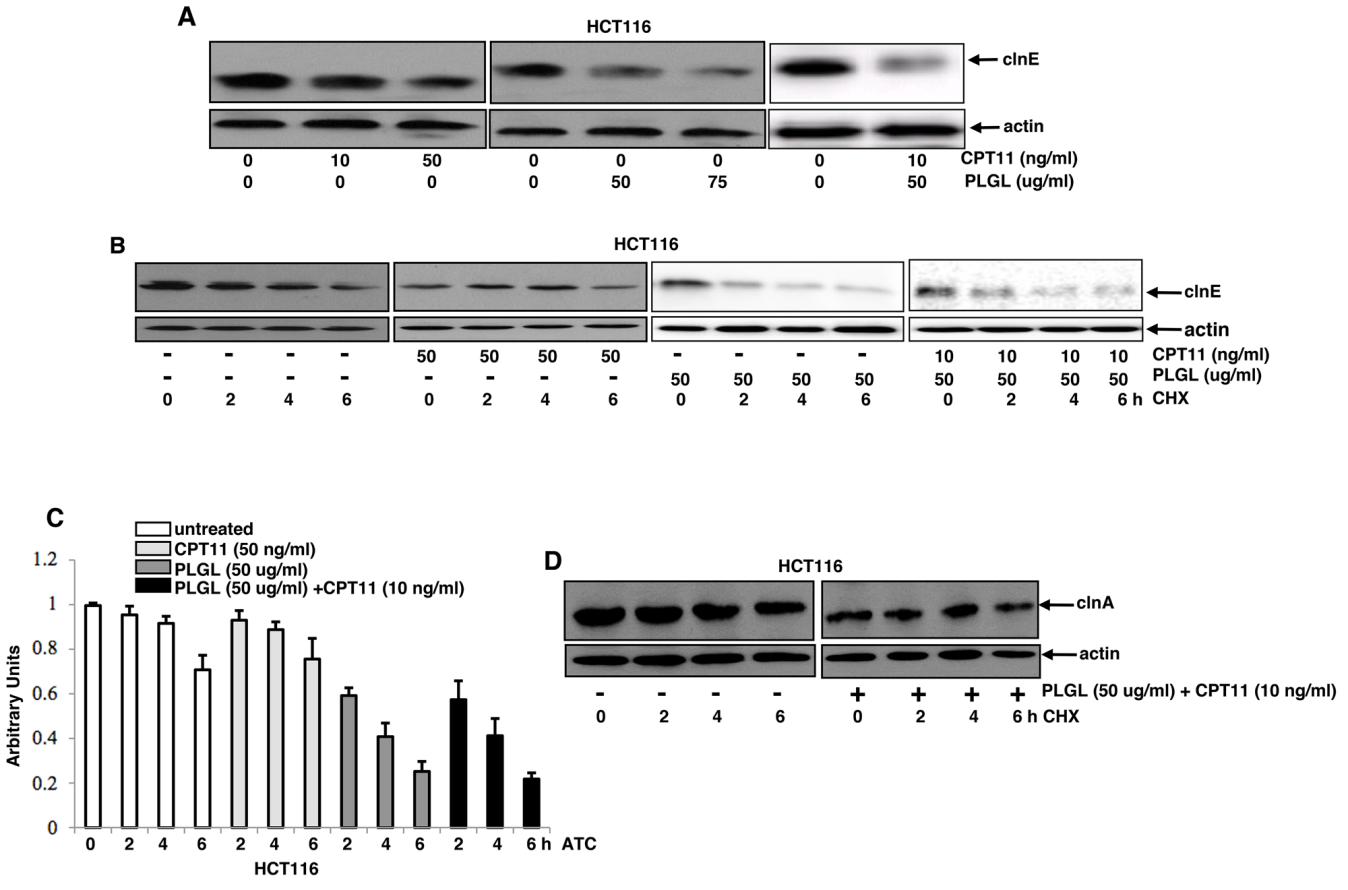
*cln E* stability was decreased at the transcription level in colon cancer cells after PLGL or its co-treatment with CPT11 **(A)** Cln E expression was tested in the cells with or without the treatments by immunoblotting. **(B)** After exposure to CHX exposure, cell lysates from untreated or treated HCT116 cells at different time points of CHX blocking were prepared and then tested for the expression of cln E by immunoblotting. **(C)** After actinomycin D (ACT) treatment, total mRNAs from untreated or treated HCT116 cells were isolated at different times and subjected to RT-PCR analysis. Error bars are SD from 5 experiments *(p*<0.001). **(D)** After CHX treatment, cell lysates from untreated or treated HCT116 cells were prepared at different time points and then tested for the expression of cln A by immunoblotting.

Because clnE is one of the key regulators of S phase, its stability was tested in our experimental setting. HCT116 cells were treated with various treatments for 2 h, and then subjected to immunoblotting (Figure 5A). The level of clnE expression in PLGL-treated HCT116 cells was decreased in a dose-dependent fashion, the reduction of which was also detected in the same cells received the co-treatment. However, CPT11 treatment at different doses did not obviously alter the levels of clnE expression. The protein stability of clnE was then tested (Figure 5B). After blocked protein synthesis by CHX, the expression of clnE in untreated HCT116 cells or the cells treated with the high dose of CPT11 (50 ng/ml) was relatively stable and started to decrease 6 h after the blocking. In comparison, clnE became unstable in PLGL- or co-treated cells, the levels of which started to decrease 2 h after blocking protein synthesis. The *clnE* gene stability was then analyzed by RT-PCR (Figure 5C). After treated with actinomycin D (ATC) to block gene transcription machinery, the level of *clnE* in PLGL- or co-treated cells, but not in untreated or CPT11-treated cells, was rapidly degraded. The protein stability of clnA was also examined (Figure 5D). The co-treatment did not affect clnA stability. The stability *clnA* at gene level in the cells was also tested, which was not changed by the co-treatment (data not shown). It seemed that PLGL specifically targeted *clnE* gene stability of colon cancer cells.

### Ectopic overexpressions of Chk1 and clnE blocked the lethal synergy induced by the co-treatment

**Figure 6:**
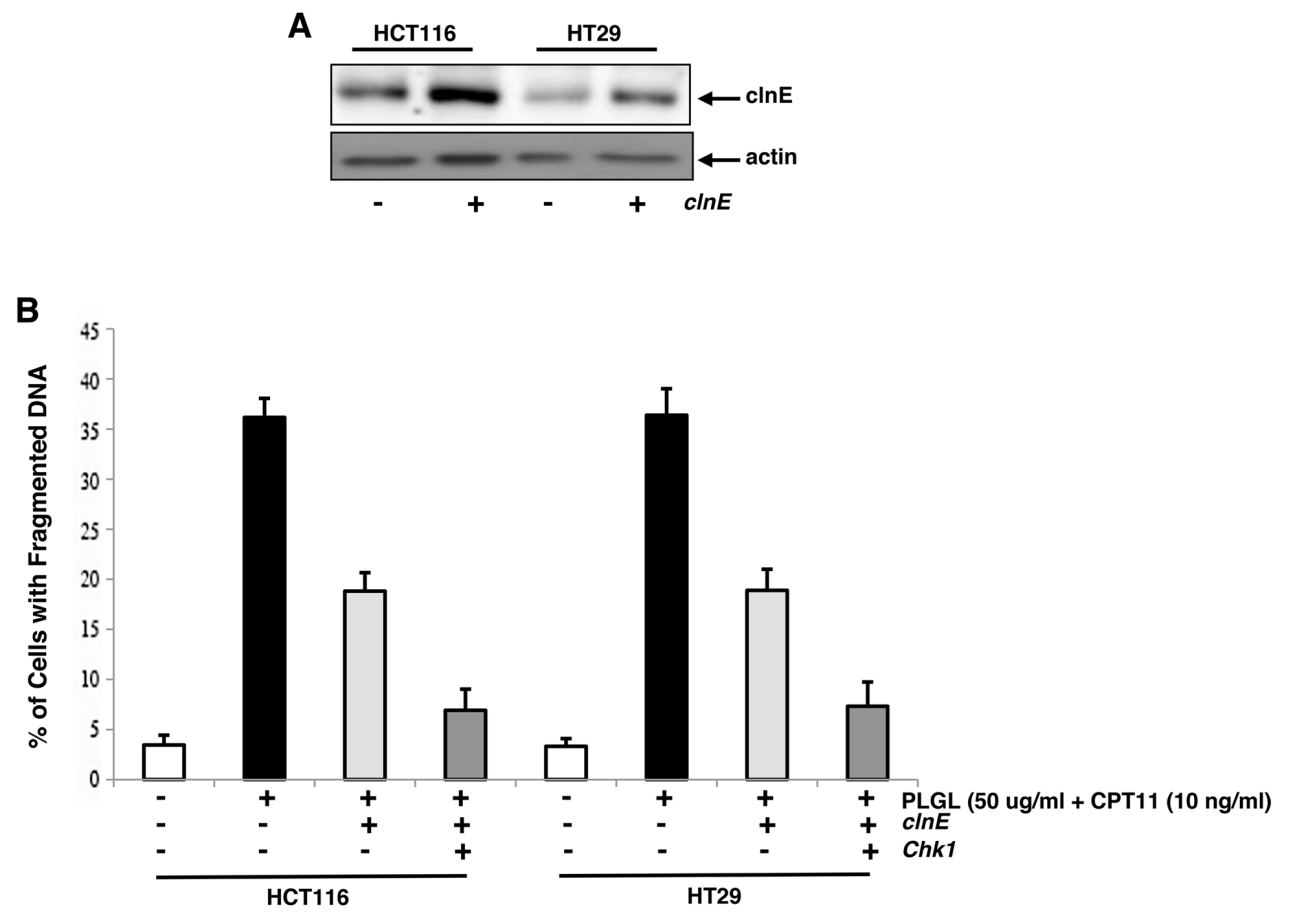
Ectopic expression of *clnE* and *Chk1* blocked co-treated colon cancer cells to undergo apoptosis **(A)** *ClnE* was introduced into HCT116 or HT29 cells and its protein expression in the cells was determined by immunoblotting. **(B)** Colon cancer cells were transfected with *clnE* or *clnE* plus *Chk1*, and then received the co-treatment for 48 h. Subsequently, DNA fragmentation assay was performed to detect the induction of apoptosis. Error bars are SD over 5 independent experiments (*p*<0.005).

In order to test the importance of Chk1 and clnE in the induction of apoptosis upon the co-treatment, *clnE* and *Chk1* were ectopically overexpressed. The expression of clnE (Figure 6A) or Chk1 (Figure 4A) in HCT116 cells transfected with the corresponding genes was analyzed by immunoblotting and Subsequently, the effects of the overexpression of *clnE* and its co-expression with *Chk1* on the induction of lethal synergy were examined in co-treated HCT116 and HT29 cells (Figure 6B). The ectopic expression of *clnE* partially suppressed apoptosis induced by the co-treatment of PLGL and CPT11. However, the co-overexpression of *clnE* and *Chk1* almost completely inhibited apoptosis induced by the co-treatment. The data suggested that Chk1 and clnE were the targets of the co-treatment in the induction of the lethal synergy.

## DISCUSSION

PL is a species of fungi that belong to the *Hymenochaetaceae Basidiomycetes*, and the polysaccharides extracted from PL (PLGL) have been widely used in Asia for treating cancers among other diseases [1–3]. However, the use of PLGL is mostly based on empirical practice, which lacks the evidence-based experimental data to support its clinical implementation. Because of the deficiency in the understanding of PLGL, we started to investigate the underlying mechanisms by which PLGL antagonizes tumorigenesis. Previously, we demonstrated that PLGL attenuated lung tumorigenesis via negatively influencing cell cycle progression [17]. In current investigation, we evaluated the effect of the combination treatment of PLGL and the low dose of CPT11 on human colon cancer cells. The concurrent treatment of PLGL and CPT11 at the sub-lethal doses induced colon cancer cells, but not immortalized colon Caco-2 cells, to undergo apoptosis. In this apoptotic process, Chk1 was phosphorylated, but rapidly degraded in the colon cancer cells. As the result, the cancer cells were accumulated in S phase of the cell cycle and subsequently underwent apoptosis. Furthermore, the treatment of PLGL specifically targeted and shortened *clnE* half-life, which functioned as an additional element to promote its lethal synergy with low dose of CPT11. Overall, our findings provide the research evidence for developing PLGL to treat cancer patients, especially colon cancer patients.

During S phase of the cell cycle, ATR/Chk1 signaling controlled proper DNA replication to avoid aberrant or multiple firings at the replication origins when the replication fork progression was impaired [22]. Based on studies in yeast and xenopus, it appeared that stalled replication forks brought the ATR-ATRIP complex into the vicinity of the human homolog of claspin (a protein first identified in xenopus egg extracts), which further triggered Chk1 activation [44]. Chkl contains several closely approximated Ser/Thr-GIn (S/T-Q) motifs. In response to DNA damage or replication stress, Chkl was phosphorylated at two key sites, Ser-317 and Ser-345, in the S/T-Q-rich domain [45, 46]. The phosphorylation of these residues relieved an internal auto-inhibitory constraint on Chkl catalytic domain [47]. It was also reported that the phosphorylation of Chk1 at its Ser-345 residue was required for switching on the ubiquitin-proteasome machinery, leading to destabilize or terminate Chk1 signaling. Therefore, a premature degradation of Chk1 in colon cancer cells co-treated with PLGL and CPT11 might be a key element for subsequent induction of S phase accumulation and subsequent induction of a crisis. The identification of proteins that bind to phosphorylated Chkl and regulate its activation vs. degradation is critical to our understanding of the regulation of the lethal synergy induced by the co-treatment of PLGL and CPT11.

CPT-based drugs target DNA replication process and have a long history for treating cancers. The idea of the development of these drugs is that cancer cells are highly proliferative with residing in S phase at any given time, and an elevated fraction of cancer cells is susceptible to killing by S-phase specific cytotoxic drugs. Fopoisomerase 7 is an enzyme that binds to double-stranded DNA, introduces a reversible single-strand break, and then relieves DNA supercoiling in the path of advancing DNA replication forks [48, 49]. When bound to CPT11, fopoisomerase 7 can still associate with and nick DNA strands, but is not able to re-ligate the nicked DNA strands. Thus, CPT11 or CPT-based drugs place a roadblock in advancing DNA replication forks, leading to fork stalling and the generation of DNA double strand breaks. At the same time, the drug also rapidly terminates Chk1 by accelerating its degradation. Via these functions, CPT11 treatment activates the Chk1-dependent checkpoint to eliminate cancer cells [50, 51]. Therefore, the sensitization of the Chkl destruction machinery operated by CPT-based drugs could be a potential strategy for developing new anti-cancer strategy. Our study demonstrated that PLGL could augment anti-colon tumor activity of the low dose of CPT11. In this process, PLGL seemed not only exploiting the property of CPT11 in the activation of Chk1 in colon cancer cells, but also increasing *clnE* degradation, both of which contribute to its synergy with CPT11. The findings suggest that PLGL strengthens replicative stress in colon tumors and increase the quality of Chk1-mediated checkpoint response to facilitate CPT11 anti-cancer efficacy.

PLGL was demonstrated to suppress lung cancer cell growth via strengthening the G_1_/S cell cycle restrictions [17]. During the G_1_/S transition, the G_1_ and S cyclins (cyclin D, E, and A) form complexes with CDKs at different time points and then phosphorylate Rb to promote cell cycle progression [52–55]. The activation of the D-type cyclins by growth factor stimulation occurs at the early stages of the G1 phase. The activity of clnE in various types of cells is mainly elicited in S phase. clnA functions mainly in the G_2_. It was demonstrated that PLGL could suppress CDKs, which then interfered with the functions of the S phase regulators [17]. Consequently, PLGL interference prevented the formation of active cyclin/CDK complexes and therefore, blocked S phase progression of cancer cells. In this study, we further demonstrated that PLGL was able to suppress clnE expression, via weakening its gene stability. As the result, PLGL treatment promoted persistent S phase accumulation of the colon cancer cells. Taken together, our data indicated that PLGL was able to upregulate CPT11 drug activity and destabilize *clnE*, which appeared activating Chk1 mediated checkpoint for eliminating colon cancer cells. The experiments to identify the mechanisms by which PLGL shortens *cln E* half-life as well as enhances CPT anti-cancer activity are in the process.

The side effects of chemotherapeutic drugs remain a major obstacle for their efficacies. The present study showed that PLGL exerted its anti-malignant growth properties via enhancing CPT11 pharmacological effect by augmenting the S phase checkpoint activity. Therefore, the combination treatment of low doses of CPT11 with PLGL can diminish its side effects and significantly benefit patients’ bodies. The synergistic effect of PLGL was also observed in its combination treatment with doxorubicin in human lung cancer cells [17]. The co-treatment of PLGL and doxorubicin at low concentrations perturbed the G_1_ restriction in the lung cancer cells. These studies presented different mechanisms operated by PLGL for generating lethal synergy with different anti-cancer drugs. Therefore, more detailed studies are required for elucidating the molecular mechanisms by which PLGL synergizes with anti-cancer agents.

## MATERIALS AND METHODS

### Cell lines and reagents

The human immortalized Caco-2 and colon cancer HCT116 or HT29 cells were purchased from American Tissue Culture Collection (Rockville, MD). All the cells were cultured in Dulbecco’s modified Eagle’s medium supplemented with 10% heat-inactivated fetal calf serum, 2 mM L-glutamine, 100 U/mL of penicillin, 100 mg/mL of streptomycin. PL powder was purchased from Panbio-Tech (Taejon, South Korea), and its polysaccharides were purified with ethanol precipitation methods followed by DEAE-cellulose and gel permeation chromatography [14, 17]. CPT11 was purchased from Sigma (St. Louis, MO).

### DNA fragmentation and cell cycle assays

A flow cytometric analysis was performed with a FACScan (Becton Dickenson, Mountain View, CA). The data analysis and display were performed with the Cell-Fit software program (Becton Dickenson). Cell-Fit provides data from the flow cytometer and real-time statistical analysis of the data. After various treatments, cells (1 x 10^6^/ml) were washed with 1 x PBS, fixed with 70% ethanol. Subsequently, cells were stained with propidium iodide (0.1 ug/ml) containing RNase at 1.5 ng/ml. The stained samples were kept at 4°C overnight before flow cytometric analysis for either measuring percentages of less G_1_ DNA contents of apoptotic cells or DNA profiles of cells in different phases of the cell cycle.

### Double thymidine block

Cells were cultured in the growth medium to 50%-60% confluency. The thymidine blocking solution (Sigma, St. Louis, MO) was then added in the cultures. After 19 h of culturing, the media of the cultures were removed. After grown in the fresh growth media for 9 h, the thymidine solution was added in the cultures that were grown for another 16 h when majority of cells were blocked in the boundary of the S phase of the cell cycle.

### Immunoblot

After the treatments, cells were washed in 1 x PBS and then lysed in the lysis buffer (50 mM Tris-HCl, pH 8.0, 150 mM NaCl, 1% Triton-X114, 0.5% sodium deoxycholate, 0.1% SDS, containing 1 mM phenylmethylsulfonyl fluoride, 1 ng/ml of aprotinin, 1 ng/ml of leupeptin, 1 mg/ml of pepstatin A) on ice for 30 min. The total protein concentrations in cell lysate were normalized. The samples were separated on a 10% SDS–PAGE gel and subsequently transferred to a nitrocellulose membrane. All the antibodies used were purchased from Cell Signaling Tec. (Beverly, MA). Each experiment was repeated at least 2 to 3 times.

### Quantitative real time-PCR

Total RNA was isolated using TRIzol reagent (Invitrogen) according to the instruction provided by the manufacturers. cDNA was prepared using 1 μg of total RNA extracted (iScript cDNA synthesis kit, Bio-Rad), and then subjected for the amplification by quantitative real time-PCR using Applied Biosystem StepOnePlus in the presence of SYBR Green JumpStart (Sigma-Aldrich). Ribosomal 18 S RNA was used as the normalization control. The n-fold change in mRNA expression was determined on the basis of the ΔΔCt value. The human *clnE* sense primer is: 5’-gtcctggctgaatgtatacatgc-3’ and the antisense primer is: 5’-cctatttgttcagacaacatggc-3’.

### Xenograft assay

Cells (1 x 10^6^) in 100 μl of PBS were inoculated into each BalB/c nude mouse. One group of mice (6 mice/group) was injected peritoneally with CPT11 (20 mg/kg) or co-injected with PLGL (60 mg/kg) plus CPT11 (8 mg/kg) right after the inoculation and subsequently administrated the inhibitor every 4 days. The sizes of the tumors were measured weekly and plotted. After the mice were sacrificed, the tumors were isolated, and the picture of some of tumors was taken. The intakes of the food and water of the animal were recorded routinely. The animal experiments were carried out according to the guidelines of the Animal Care and Use Committees of the Institute.

### Statistical analysis

Statistical analysis was performed using a two-tailed Student’s *t* test for comparison of two groups or a one-way analysis of variance for comparison of more than two groups followed by Tukey’s multiple comparison tests. Tumor-free probabilities were estimated using Kaplan-Meier method and were compared among groups. Standard deviations are displayed in the figures. A p value <0.05 was considered significant.
